# Prognostic Value of Chemotherapy-Induced Neutropenia at the First Cycle in Invasive Breast Cancer

**DOI:** 10.1097/MD.0000000000003240

**Published:** 2016-04-01

**Authors:** Rui-Min Ma, Chuan-Zhi Chen, Wei Zhang, Jie You, Du-Ping Huang, Gui-Long Guo

**Affiliations:** From the Department of Oncology, the First Affiliated Hospital of Wenzhou Medical University, Wenzhou, China.

## Abstract

Chemotherapy-induced neutropenia (CIN) was the most apparent side effects of bone marrow suppression with adjuvant chemotherapy. Recently, several studies revealed that CIN may predict better outcomes. However, the researches upon breast cancer were still indefinite.

We reviewed the female patients with pathologically diagnosed invasive breast cancer at the First Affiliated Hospital of Wenzhou Medical University, between Jan 2008 and Dec 2010. The lowest neutrophil counts in the second week after the first cycle of chemotherapy were collected. Clinicopathological characteristics and survival rates were compared and analyzed between the CIN group and non-CIN group.

The median follow-up time was 62 months. The differences of over-all survival and local recurrence-free survival between the 2 groups were nonsense (*P* = 0.938, *P* *=* 0.695, respectively). But the disease-free survival and distant metastasis-free survival of the CIN group were statically significantly better (HR = 0.391, *P* *=* 0.009, and HR = 0.315, *P* *=* 0.005, respectively). The bone metastasis-free survival may be responsible for the differences (HR = 0.469, *P* *=* 0.005). Subgroup analyses showed the CIN may predict lower bone metastases rates with ER positive status, premenopause or younger age (≤ 40) (*P* *=* 0.002, *P* *=* 0.004, and *P* *=* 0.0001, respectively). Cox analysis showed younger ages, N staging, and the presence of CIN were associated with bone metastasis-free survival independently adjusting to peritumoral vascular invasion (*P* < 0.05).

CIN may predict a decreased recurrence risk of breast cancer, especially bone metastases.

## INTRODUCTION

Breast cancer (BC) is regarded as the most common malignancy among women worldwide.^1^ BC is the first leading cause of female cancer death among those aged 20 to 59-year old.^2^ As is recognized, the best way to cure for BC upon diagnosis is surgery. Usually, there involves chemotherapy, which used to consolidate the achievements. Though taking neoadjuvant or adjuvant chemotherapy improves over-all survival (OS) and recurrence-free survival, the side effects cannot be ignored.^3–5^

Chemotherapy-induced neutropenia (CIN) is the most serious hematologic toxicity of cancer chemotherapy and is defined as 4 grades in accordance with National Cancer Institute Common toxicity Criteria (NCI-CTC).^6,7^ All patients treated with chemotherapy are at risk for CIN,^6^ which occurs most frequently during the first cycle.^8,9^ It is dangerous and life-threatening when fever neutropenia arises.^6^

Studies indicate that CIN predicted better outcomes in cancers such as lung cancer, advanced gastric cancer, and metastatic colorectal cancer.^10–12^ However, the improved outcomes in BC are indefinite. Ishitobi et al^13^ found that only distant disease-free survival was better in BC with neoadjuvant CIN. Meanwhile, Han et al^14^ revealed only better OS in BC with adjuvant CIN.

The purpose of this study was to investigate the deep relationship between CIN and survival of BC, and thence to figure out whether CIN could be used as an indicator of prognosis and guide for clinic work.

### METHODS

We reviewed female patients with pathologically diagnosed invasive BC (T1-4 N0-3 M0), who were treated at the First Affiliated Hospital of Wenzhou Medical University in China, between January 2008 and December 2010. Four hundred ten consecutive patients were finally enrolled with exclusion criteria as follows: unknown immunohistochemical (IHC) results or indefinite human epidermal growth factor receptor-2 (HER-2) status, bilateral invasive BC or noninvasive BC, history of abnormal bone marrow function, history of chemotherapy without NCCN guidelines or insufficient course, taking neoadjuvant chemotherapy, and no history of blood routine test (BRT) during the second week after adjuvant chemotherapy. Demographic, pathologic, and laboratory data of all patients were collected from electronic medical records. The research protocol was approved by the Ethics Committee of the First Affiliated Hospital of Wenzhou Medical University, and written informed consents were obtained from every patient. They were followed up till May 2015 to obtain survival information.

Patients took breast conserving surgery within their own command when technically and cosmetically feasible. The others underwent radical surgeries. Axillary dissection was carried out when sentinel lymph node was positive.

Pathological results were evaluated by more than 2 associate chief physicians, according to the WHO classification. The IHC standard referred to St. Gallen version 2013: estrogen receptor (ER) positive defined ≥1%; progesterone receptor (PR) positive meant ≥20%; and HER-2 over-expression considered complete and intense membrane staining of at least 10% of neoplastic cells (3+). Tumors showing weak-to-moderate circumferential membrane immunoreactivity (2+) of HER-2 were further subjected to fluorescence in situ hybridization assays.

Endocrine therapy for more than 5 years was recommended to ER-positive patients. Patients with HER-2 over-expression were prescribed to receive Herceptin, a target therapy, after chemotherapy. (Among 238 ER-positive patients, 11 obtained the IHC rates above 1% and less than 10%. They were reassessed before taking the endocrine therapy because of the changed criterion in 2010.).

### Chemotherapy and Supportive Treatment

Adjuvant chemotherapy was performed in 10 days after operation. The patients had received 6 cycles of intravenous FEC (5-fluorouracil 600 mg/m^2^, epirubicin 90 mg/m^2^, and cyclophosphamide 600 mg/m^2^), TEC (docetaxel 75 mg/m^2^, epirubicin 60 mg/m^2^, and cyclophosphamide 500 mg/m^2^), or TC (docetaxel 75 mg/m^2^ and cyclophosphamide 600 mg/m^2^).

Granulocyte-colony-stimulating factor (G-CSF) was used in grade 4 neutropenia or febrile neutropenia. The next cycle of chemotherapy was delayed until recovery for a neutrophil count of 1.5 × 10^9^/L from neutropenia or any other significant hematologic toxicity.

### Assessment of Neutropenia

Patients were suggested to take BRTs after chemotherapy on day 7, day 10, day 14, and the day before the next cycle (day 20 or day 21). Laboratory data, obtained after the first cycle with the lowest neutrophil count, were collected before taking G-CSFs. Neutropenia was graded in accordance with NCI-CTC version 4.0 (grade 0 equates to within normal limits; grade 1 equates to a neutrophil count of between 1.5 and 2.0 × 10^9^/L; grade 2 equates to a neutrophil count of between 1.0 and 1.5 × 10^9^/L; grade 3 equates to a neutrophil count of between 0.5 and 1.0 × 10^9^/L; and grade 4 equates to a neutrophil count below 0.5 × 10^9^/L).

### Follow-Up

All patients underwent physical examinations, blood tests, breast ultrasound, and chest X-rays every 3 to 6 months for the first 5 years postoperatively, and they were followed up every year thereafter. Computed tomography or emission computed tomography was used when metastasis was suspected. And the metastatic area was defined as the first diagnosed one.

OS was defined as the time from surgery to death. Disease-free survival (DFS) was defined as the time from surgery to local, regional, or distant treatment failure. Local recurrence-free survival (LRFS) was defined as the time from surgery to local or regional lymph-node recurrence without distant issues. Distant metastasis-free survival (DMFS) was defined as the time from surgery to distant metastasis, consisting of bone metastasis-free survival (BMFS) and visceral metastasis-free survival (VMFS).

### Statistical Analysis

Statistical analysis was performed using IBM SPSS Statistic version 21.0 (SPSS Inc., Chicago, IL, USA) and GraphPad Prism version 6.01 (GraphPad Software Inc., La Jolla, CA, USA). Kolmogorov–Smirnov test was used to test for normality within continuous data. Those with normal distribution were expressed as the mean ± standard deviation and then compared using a Student *t* test. Otherwise, continuous data with abnormal distribution were expressed as the median (range) and then compared using the Wilcoxon rank-sum test. Categorical variables were compared using the *χ*^2^ test or Fisher exact test as appropriate. Censoring time was defined as the last follow-up time. Kaplan–Meier survival curves with log-rank tests and Cox proportional hazard regression analyses were used to compare the survival rates with clinical and pathologic factors. Variables with *P* <0.05 in univariate Cox regression analysis were progressed to multivariate analysis by forward stepwise selection. A value of *P* <0.05 was considered statistically significant and all *P* values were 2-tailed.

## RESULTS

### Clinicopathologic Characteristics

A total of 410 cases were enrolled in this study. The baseline characteristics of the invasive BC patients with and without neutropenia are listed in Table 1. Among these patients, 330 met the criteria for neutropenia, including 44 of grade 1, 74 of grade 2, 101 of grade 3, and 111 of grade 4. By comparison, there were no statistically significant differences between the 2 groups in terms of age, menopausal status, lymphocytes or palate counts, pathological T or N staging, receptor conditions, and the presence of peritumoral vascular invasion (PVI). The modality of treatment was also similar between the 2 groups. However, the CIN group exhibited significantly lower leukocyte count and neutrophil count before treatment (*P* < 0.001). The rates of CIN were higher with anthracycline-based regimens (*P* < 0.001).

**TABLE 1:**
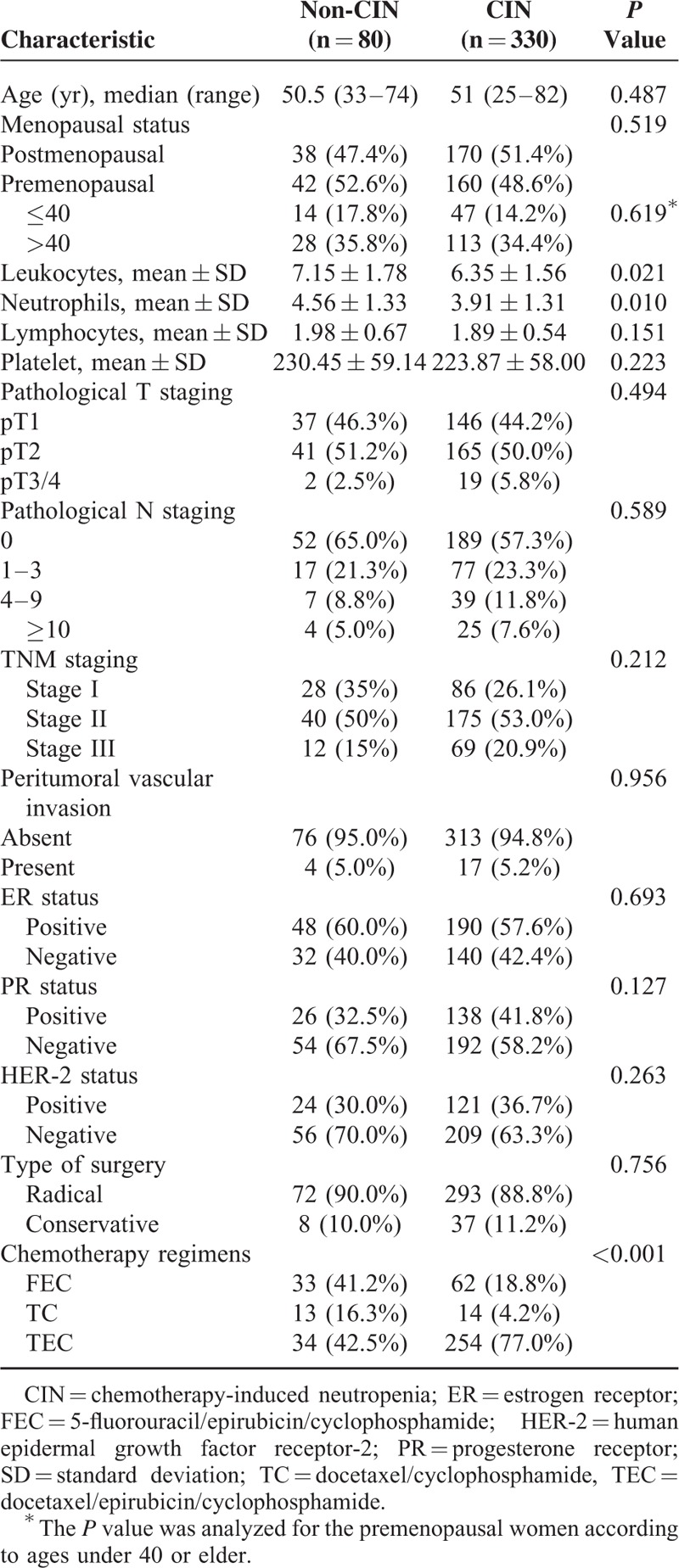
Clinicopathologic Characteristics of Patients With or Without Involvement of CIN

In addition, patients with more advanced TNM stages or pathological N staging showed higher rates of low-grade CIN (*P* = 0.005 and *P* = 0.017, respectively in Supplementary Table 1). And there were more TEC regimens exhibiting lower-grade CIN, compared with other regimens (*P* < 0.001 in Supplementary Table 1).

### Overall Survival Analysis

**FIGURE 1:**
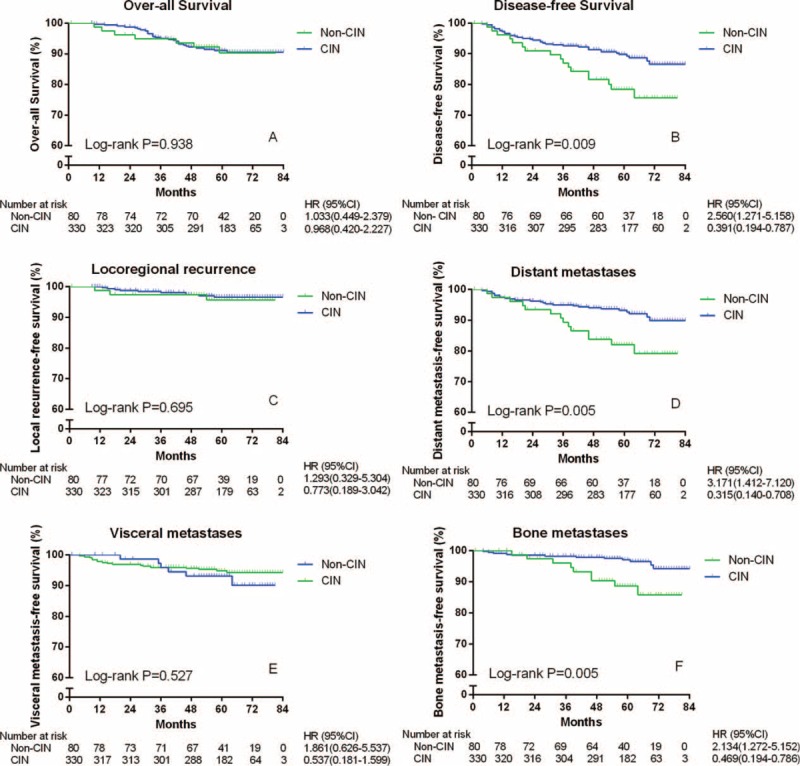
Survival and clinical outcomes of 410 women with invasive breast cancer according to the presence or absence of CIN. CI = confidence interval; CIN = chemotherapy-induced neutropenia; HR = hazard ratio.

A total of 26 patients (6.34%) were lost to follow-up. The median follow-up time of the cohort was 62 months. During the follow-up period, 36 patients died of BC. The 5-year OS rates were 91.12% in the non-CIN group and 90.28% in the CIN group. As shown in Figure 1A, no difference in OS was found between the patients with CIN and those without CIN (*P* *=* 0.938).

### Disease-Free Survival Analysis

During the follow-up period, freedom from recurrence was observed in 63 patients in the non-CIN group and 295 in the CIN group. As shown in Figure 1B, the difference between the 2 curves was statistically significant (*P* = 0.009, hazard ratio [HR] = 0.391, 95% confidence interval [CI] 0.194–0.787). The cumulative 2- and 5-year DFS rates in the CIN group were 94.85% and 90.61%, respectively. And the rates in the non-CIN group were 91.25% and 80.00%, accordingly. Only the latter was significantly different between the 2 groups (*P* = 0.214 and *P* = 0.006, respectively).

During the follow-up period, 3 patients in the non-CIN group and 10 patients in the CIN group were found to demonstrate loco-regional recurrence. Meanwhile, 14 and 25 patients suffered from distant metastases in the non-CIN group and the CIN group, respectively. As shown in Figure 1C, there was no significant difference in the rate of loco-regional recurrence-free survival between the 2 groups (*P* = 0.695). However, as shown in Figure 1D, the DMFS rate in the CIN group was significantly higher than that in the non-CIN group (HR = 0.315, 95% CI 0.014–0.708, *P* = 0.005).

Among the cases with distant metastases, the numbers of patients diagnosed with visceral metastases and bone metastases between the 2 groups (non-CIN versus CIN) were 6 versus 17, and 9 versus 12, respectively. As shown in Figure 1E, there was no difference in the rate of VMFS between the groups (*P* *=* 0.527). Nevertheless, the rate of BMFS in the CIN group was significantly higher than that in the non-CIN group (HR = 0.469, 95% CI 0.194–0.786, *P* *=* 0.005) (Figure 1F).

In addition, no differences in the rates of DFS, DMFS, and BMFS were found between the mild-CIN group and the severe-CIN group (*P* > 0.05; Supplementary Figures 1B, 1D, and 1F); meanwhile, both groups showed significant differences from the non-CIN group (*P* < 0.05; HR < 1; Supplementary Figures 1B, 1D, and 1F).

### Subgroup Analyses

**FIGURE 2:**
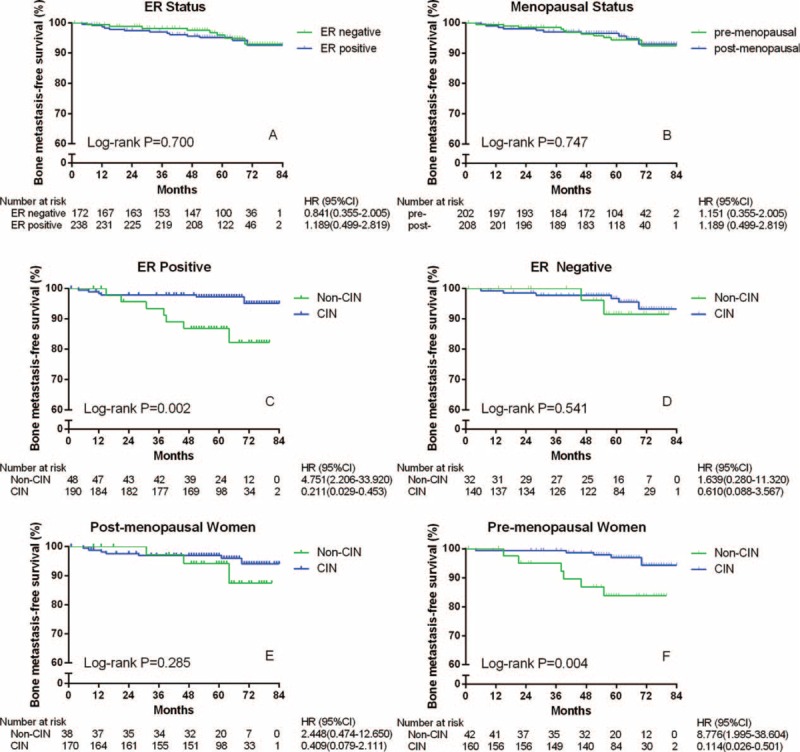
Bone metastasis-free survival of 410 women with invasive breast cancer according to ER status and menopausal condition, and subgroup analyses with ER status or menopausal condition according to the presence or absence of CIN. CI = confidence interval; CIN = chemotherapy-induced neutropenia; ER = estrogen receptor; HR = hazard ratio; post- = postmenopausal; pre- = premenopausal.

In the subgroup analyses, the bone metastasis rates were reevaluated, adjusting for ER presence and menopausal status. As shown in Figure 2A, a trend was found showing higher bone metastasis rates in the ER-positive group than in the ER-negative group. However, the *P* value was apparently not significant (*P* *=* 0.700). In the ER-positive subgroup (Figure 2C), the patients with CIN exhibited extremely higher BMFS rates than did those without CIN (HR = 0.211, 95% CI 0.029–0.453, *P* *=* 0.002). Meanwhile, in the ER-negative subgroup (Figure 2D), there was no difference between the patients with and without CIN (*P* *=* 0.541).

**FIGURE 3:**
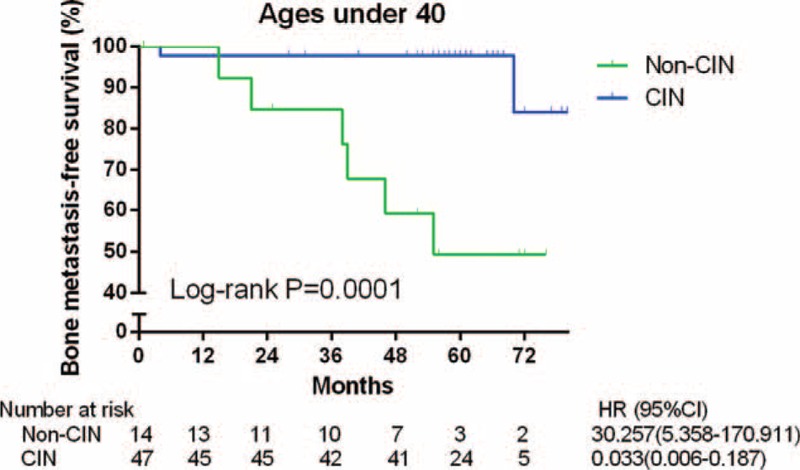
Bone metastasis-free survival of 61 women with invasive breast cancer and ages under 40-year old according to the presence or absence of CIN. CI = confidence interval; CIN = chemotherapy-induced neutropenia; HR = hazard ratio.

As shown in Figure 2B, the menopausal status caused no difference (*P* *=* 0.747). In postmenopausal women (Figure 2E), the difference between the patients with and without CIN was not significant (*P* *=* 0.285). However, in premenopausal women (Figure 2F), patients with CIN obtained significantly higher BMFS rates than did those without CIN (HR = 0.114, 95% CI 0.026–0.501, *P* *=* 0.004). When carried out among the younger premenopausal women (≤40 years of age), the differences were still significant (Figure 3, *P* < 0.001).

### Cox Analyses

Cox proportional hazard models were used to identify the variables associated with OS, DFS, and BMFS. These variables are presented in Table 2.

**TABLE 2:**
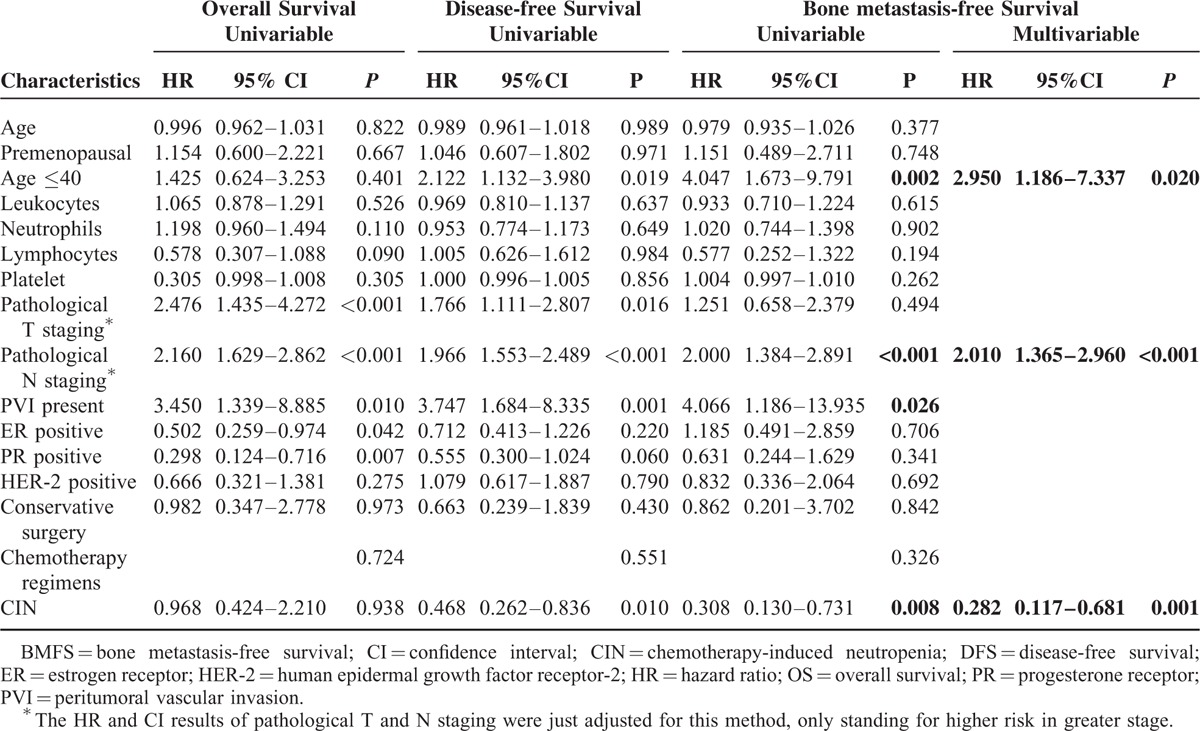
Cox Proportional Hazards Regression Models of Risk Factors Associated With OS, DFS, and BMFS Among Breast Cancer Patients (n = 410)

In univariate analysis, age, premenopausal status, HER-2-positive status, conservative surgery, and chemotherapy regimens, as well as counts of leukocytes, neutrophils, lymphocytes, and platelets were not significant predictive factors for the prognosis of OS, DFS, or BMFS in the patients with invasive BC. Pathological T staging was significantly related to OS and DFS. The ER- or PR-positive status was a significant predictive factor only for OS and not for DFS or BMFS. The presence of CIN and age under 40 were only inversely associated with DFS and BMFS. PVI and the pathological N staging were significant prognostic factors for all cases.

In the multivariate Cox analysis of BMFS, the presence of CIN, pathological N staging, and age under 40 were independently predictive risk factors for the prognosis of patients with invasive BC (*P* < 0.05, Table 2).

## DISCUSSION

Neutropenia is a common side effect of chemotherapy in adjuvant treatment of BC. This condition is typically regarded as a dose-limiting toxicity and a reason for dose reduction. CIN occurs most frequently during the first cycle of chemotherapy.^8,9^ The absolute neutrophil count often increases on the second week during the first cycle.^15–17^ More aggressive regimens, which are used in more advanced patients, may lead to higher rates of CIN.^18^ However, in our study, the aforementioned regimens did not induce a significant prognostic difference.

Moreover, the presence of CIN in patients with postoperatively invasive BC, as a response of patient condition to chemotherapy, showed an independently predictive significance of prognosis. Both mild and severe CIN were associated with a risk of recurrence (HR = 0.343, 95% CI 0.153–0.770, *P* = 0.006 and HR = 0.538, 95% CI 0.291–0.989, *P* = 0.043, respectively, in Supplement Figure 1), particularly for distant metastases (*P* = 0.005 and *P* = 0.043, respectively) but not mortality. And the differences appeared after the second year. Additionally, the difference in VMFS between the patients with and without CIN was not significant, whereas the difference in BMFS was statistically significant. This finding can be mainly responsible for the association between distant metastases and CIN, even after adjusting for potential clinicopathogical variables (HR = 0.282, 95% CI 0.117–0.681, *P* = 0.005). The following median survival reported was approximately 2 years and the 5-year survival rate was almost 40% after diagnosis of bone metastases.^19^ Thus, the median survival of our patients with bone metastases would be possibly 63 months, which is longer than the median of the follow-up time (62 months), according to the median BMFS of this research (39 months). Thus, the disparity of OS and DFS, which was almost the same to the results of the 62-month Austrian Breast and Colorectal Cancer Study Group Trial-12 (ABCSG-12), could be explained in a sense.^20^ However, bone metastases still played a very important role in the procedure, leading patients to death, and with more than 10 times of HR.^21^ In the first year, skeletal-related events (SREs) are expected to follow because of osteolysis.^22^ SREs, which include pathological fractures, spinal cord compression, and severe pain requiring radiotherapy or surgery, would further increase mortality and reduce the quality of life as well.^21,23^

The bones have been proven to be the most preferential metastatic target sites for BC.^24,25^ “Seed and soil” theory is well acknowledged for bone metastases: circulating cancer cells (seeds) can accomplish metastasis in the organs, of which microenvironment (soil) is advantageous for their growth.^25,26^ PVI was associated with cancer cell invasion from primary location, and was proven to be a high-risk predictor of the BMFS in our study (HR = 4.066, 95% CI 1.186–13.935, *P* = 0.026). Immobilized growth factors fertilized the bone marrow, including the transforming growth factor β (TGF-β), insulin-like growth factors (IGFs), fibroblast growth factors (FGFs), and platelet-derived growth factors (PDGFs).^25^ The genes of chemokine receptor-4 (CXCR4), parathyroid hormone-like hormone (PTHLH), interleukin-11 (IL11), matrix metalloproteinase-1 (MMP1), and osteopontin (OPN) have been proven capable of promoting bone metastases by interacting with the aforementioned growth factors.^27^ The positive expression of ER, which was evaluated in primary BC, was firmly connected to bone metastases through the reactions between the genes and growth factors.^25,28^ In the current study, a trend of worse prognosis was observed during the first 5 years, almost the median follow-up time. Considering the number of subjects at risk, this trend may become a more significant result in another following 5 years (Figure 2A). The subgroup analysis, ruling out the effect of the ER status, indicated that CIN might be a protective predictor of bone metastases only to ER-positive BC. Maybe, chemotherapy-induced bone marrow suppression, including CIN, could decrease the quantity and activity of growth factors, which can interact with ER and make the cancer cells more aggressive.^25,28^

Premenopausal status has been considered a high-risk predictor of bone metastases in patients with BC.^29^ The increased estrogen concentration in premenopausal patients was one of the possible reasons for recurrence. And the usage of tamoxifen among premenopausal women can accelerate osteolytic metabolism and cause significant bone loss, which may lead to bone metastases.^30^ In the present study, the BMFS of premenopausal patients with CIN improved better (Figures 2E and F). However, no significant difference between the 2 menopausal statuses was observed. Interestingly, women, under 40 years of age, showed an obviously high risk of bone metastases even after multivariate analysis and had got great benefit from CIN (Table 2 and Figure 3). They were found unable to prevent the bone metastases by treating with Zoledronic acid.^20^ Perhaps, the high level of estrogen before 40-year old, which could not be suppressed completely by ovarian function suppression, may lead to more aggressive results.^20,31^ What is more, young patients were more frequently diagnosed as having triple negative BC (TNBC) with a high prevalence of BRCA-1 mutations, especially younger than 40.^32,33^ And there were more bone metastases than other distant recurrences observed in Chinese people with TNBC.^34^

Besides, neutrophil counts of pretreatment in patients with CIN were lower than the non-CIN group (Table 1, *P* *=* 0.010). It is said that the neutrophil count is capable of predicting CIN.^35^

Nevertheless, our study has several limitations. First, the criterion of nonneutropenia, which is above lowest normal index, differed from labs. Second, the BRTs taken uncontinuously in day 7, day 10, and day 14 of enrolled patients might not obtain the lowest neutrophil counts. Third, the sample size and follow-up time limited us to getting more significantly prognostic results or doing further subgroup analyses. Last, the retrospective design of our study might lead to selection bias. Future prospective studies are needed to address the influence of races or ethnic groups on the conclusions of the current study.

In summary, our current study suggests that CIN of the first cycle may predict a decreased recurrence risk in patients with invasive BC, especially the bone metastases, regardless of the differences among chemotherapy regimens. Non-CIN, age under 40, and high grade of N staging were all independently increased factors of bone metastases. And the presence of CIN would be a significant predictor of decreased bone metastases only when patients were performed positive ER status. Hence, further investigations are needed to elucidate the underlying mechanisms. More attention should be given to BC patients without CIN during their first cycles, and they should be provided with appropriate treatment in the future.

## Supplementary Material

Supplemental Digital Content

## References

[1] Ferlay J Soerjomataram I Dikshit R Cancer incidence and mortality worldwide: sources, methods and major patterns in GLOBOCAN 2012. *Int J Cancer* 2015; 136:E359–E386. 25220842 10.1002/ijc.29210

[2] Siegel RL Miller KD Jemal A Cancer statistics, 2015. *CA Cancer J Clin* 2015; 65:5–29. 25559415 10.3322/caac.21254

[3] Early Breast Cancer Trialists’ Collaborative G Peto R Davies C Comparisons between different polychemotherapy regimens for early breast cancer: meta-analyses of long-term outcome among 100,000 women in 123 randomised trials. *Lancet* 2012; 379:432–444. 22152853 10.1016/S0140-6736(11)61625-5 PMC3273723

[4] Early Breast Cancer Trialists’ Collaborative G Effects of chemotherapy and hormonal therapy for early breast cancer on recurrence and 15-year survival: an overview of the randomised trials. *Lancet* 2005; 365:1687–1717. 15894097 10.1016/S0140-6736(05)66544-0

[5] Partridge AH Burstein HJ Winer EP Side effects of chemotherapy and combined chemohormonal therapy in women with early-stage breast cancer. *J Natl Cancer Inst Monogr* 2001; 135–142. 11773307 10.1093/oxfordjournals.jncimonographs.a003451

[6] Crawford J Dale DC Lyman GH Chemotherapy-induced neutropenia: risks, consequences, and new directions for its management. *Cancer* 2004; 100:228–237. 14716755 10.1002/cncr.11882

[7] Kaba H Fukuda H Yamamoto S Ohashi Y [Reliability at the National Cancer Institute-Common Toxicity Criteria version 2.0]. *Gan To Kagaku Ryoho* 2004; 31:1187–1192. 15332541

[8] Lyman GH Kuderer NM Crawford J Predicting individual risk of neutropenic complications in patients receiving cancer chemotherapy. *Cancer* 2011; 117:1917–1927. 21509769 10.1002/cncr.25691 PMC3640637

[9] Lopez-Pousa A Rifa J Casas de Tejerina A Risk assessment model for first-cycle chemotherapy-induced neutropenia in patients with solid tumours. *Eur J Cancer Care (Engl)* 2010; 19:648–655. 20088918 10.1111/j.1365-2354.2009.01121.x PMC3082427

[10] Chen Z Chen W Wang J Pretreated baseline neutrophil count and chemotherapy-induced neutropenia may be conveniently available as prognostic biomarkers in advanced gastric cancer. *Intern Med J* 2015; 45:854–859. 25871806 10.1111/imj.12786

[11] Rambach L Bertaut A Vincent J Prognostic value of chemotherapy-induced hematological toxicity in metastatic colorectal cancer patients. *World J Gastroenterol* 2014; 20:1565–1573. 24587632 10.3748/wjg.v20.i6.1565 PMC3925865

[12] Kishida Y Kawahara M Teramukai S Chemotherapy-induced neutropenia as a prognostic factor in advanced non-small-cell lung cancer: results from Japan Multinational Trial Organization LC00-03. *Br J Cancer* 2009; 101:1537–1542. 19862000 10.1038/sj.bjc.6605348 PMC2778518

[13] Ishitobi M Komoike Y Motomura K Prognostic significance of neutropenia on day one of anthracycline-based neoadjuvant chemotherapy in operable breast cancer. *Oncology* 2010; 78:213–219. 20424493 10.1159/000313702

[14] Han Y Yu Z Wen S Prognostic value of chemotherapy-induced neutropenia in early-stage breast cancer. *Breast Cancer Res Treat* 2012; 131:483–490. 21971729 10.1007/s10549-011-1799-1

[15] Lindman H Astrom G Ahlgren J Individually tailored toxicity-based 5-fluorouracil, epirubicin and cyclophosphamide (FEC) therapy of metastatic breast cancer. *Acta Oncol* 2007; 46:165–171. 17453364 10.1080/02841860600871087

[16] Martin M Lluch A Segui MA Toxicity and health-related quality of life in breast cancer patients receiving adjuvant docetaxel, doxorubicin, cyclophosphamide (TAC) or 5-fluorouracil, doxorubicin and cyclophosphamide (FAC): impact of adding primary prophylactic granulocyte-colony stimulating factor to the TAC regimen. *Ann Oncol* 2006; 17:1205–1212. 16766587 10.1093/annonc/mdl135

[17] del Giglio A Eniu A Ganea-Motan D XM02 is superior to placebo and equivalent to Neupogen in reducing the duration of severe neutropenia and the incidence of febrile neutropenia in cycle 1 in breast cancer patients receiving docetaxel/doxorubicin chemotherapy. *BMC Cancer* 2008; 8:332. 19014494 10.1186/1471-2407-8-332 PMC2628928

[18] Mackey JR Martin M Pienkowski T Adjuvant docetaxel, doxorubicin, and cyclophosphamide in node-positive breast cancer: 10-year follow-up of the phase 3 randomised BCIRG 001 trial. *Lancet Oncol* 2013; 14:72–80. 23246022 10.1016/S1470-2045(12)70525-9

[19] Kozlow W Guise TA Breast cancer metastasis to bone: mechanisms of osteolysis and implications for therapy. *J Mammary Gland Biol Neoplasia* 2005; 10:169–180. 16025223 10.1007/s10911-005-5399-8

[20] Gnant M Mlineritsch B Stoeger H Adjuvant endocrine therapy plus zoledronic acid in premenopausal women with early-stage breast cancer: 62-month follow-up from the ABCSG-12 randomised trial. *Lancet Oncol* 2011; 12:631–641. 21641868 10.1016/S1470-2045(11)70122-X

[21] Yong M Jensen AO Jacobsen JB Survival in breast cancer patients with bone metastases and skeletal-related events: a population-based cohort study in Denmark. *Breast Cancer Res Treat* 2011; 129:495–503. 21461730 10.1007/s10549-011-1475-5

[22] Jensen AO Jacobsen JB Norgaard M Incidence of bone metastases and skeletal-related events in breast cancer patients: a population-based cohort study in Denmark. *BMC Cancer* 2011; 11:29. 21261987 10.1186/1471-2407-11-29 PMC3037922

[23] Matza LS Chung K Van Brunt K Health state utilities for skeletal-related events secondary to bone metastases. *Eur J Health Econ* 2014; 15:7–18. 23355121 10.1007/s10198-012-0443-2 PMC3889679

[24] Quayle L Ottewell PD Holen I Bone metastasis: molecular mechanisms implicated in tumour cell dormancy in breast and prostate cancer. *Curr Cancer Drug Targets* 2015; 15:469–480. 25968899 10.2174/1568009615666150506092443

[25] Zhang Y Ma B Fan Q Mechanisms of breast cancer bone metastasis. *Cancer Lett* 2010; 292:1–7. 20006425 10.1016/j.canlet.2009.11.003

[26] Ribelles N Santonja A Pajares B The seed and soil hypothesis revisited: current state of knowledge of inherited genes on prognosis in breast cancer. *Cancer Treat Rev* 2014; 40:293–299. 24112814 10.1016/j.ctrv.2013.09.010

[27] Kang Y Siegel PM Shu W A multigenic program mediating breast cancer metastasis to bone. *Cancer Cell* 2003; 3:537–549. 12842083 10.1016/s1535-6108(03)00132-6

[28] Wang J Jarrett J Huang CC Identification of estrogen-responsive genes involved in breast cancer metastases to the bone. *Clin Exp Metastasis* 2007; 24:411–422. 17593529 10.1007/s10585-007-9078-6

[29] Braun S Vogl FD Naume B A pooled analysis of bone marrow micrometastasis in breast cancer. *N Engl J Med* 2005; 353:793–802. 16120859 10.1056/NEJMoa050434

[30] Wright LE Guise TA The microenvironment matters: estrogen deficiency fuels cancer bone metastases. *Clin Cancer Res* 2014; 20:2817–2819. 24803577 10.1158/1078-0432.CCR-14-0576 PMC4061908

[31] Hill N Madarnas Y Failure of ovarian ablation with goserelin in a pre-menopausal breast cancer patient resulting in pregnancy: a case report and review of the literature. *Breast Cancer Res Treat* 2011; 129:265–268. 21541703 10.1007/s10549-011-1542-y

[32] Azim HA Jr Michiels S Bedard PL Elucidating prognosis and biology of breast cancer arising in young women using gene expression profiling. *Clin Cancer Res* 2012; 18:1341–1351. 22261811 10.1158/1078-0432.CCR-11-2599

[33] Criscitiello C Azim HA Jr Schouten PC Understanding the biology of triple-negative breast cancer. *Ann Oncol* 2012; 23 Suppl 6:vi13–vi18. 23012296 10.1093/annonc/mds188

[34] Lin Y Yin W Yan T Site-specific relapse pattern of the triple negative tumors in Chinese breast cancer patients. *BMC Cancer* 2009; 9:342. 19778431 10.1186/1471-2407-9-342 PMC2760577

[35] Kwon WA Oh TH Lee JW Park SC Predictive factors for neutropenia after docetaxel-based systemic chemotherapy in Korean patients with castration- resistant prostate cancer. *Asian Pac J Cancer Prev* 2014; 15:3443–3446. 24870736 10.7314/apjcp.2014.15.8.3443

