# Supplementary material for: Prognostic Value of Chemotherapy-Induced Neutropenia at the First Cycle in Invasive Breast Cancer
Source: Medicine (Baltimore). 2016 Apr 1;95(13):e3240. doi: 10.1097/MD.0000000000003240 (PMC4998558; doi:10.1097/MD.0000000000003240)
Supplement: Supplemental Digital Content [file medi-95-e3240-s001.doc]

Supplementary Figure.1 Survival and clinical outcomes of 410 women with invasive breast cancer according to the degree of CIN. CIN=Chemotherapy-induced neutropenia; HR=Hazard ratio; CI=Confidence interval.


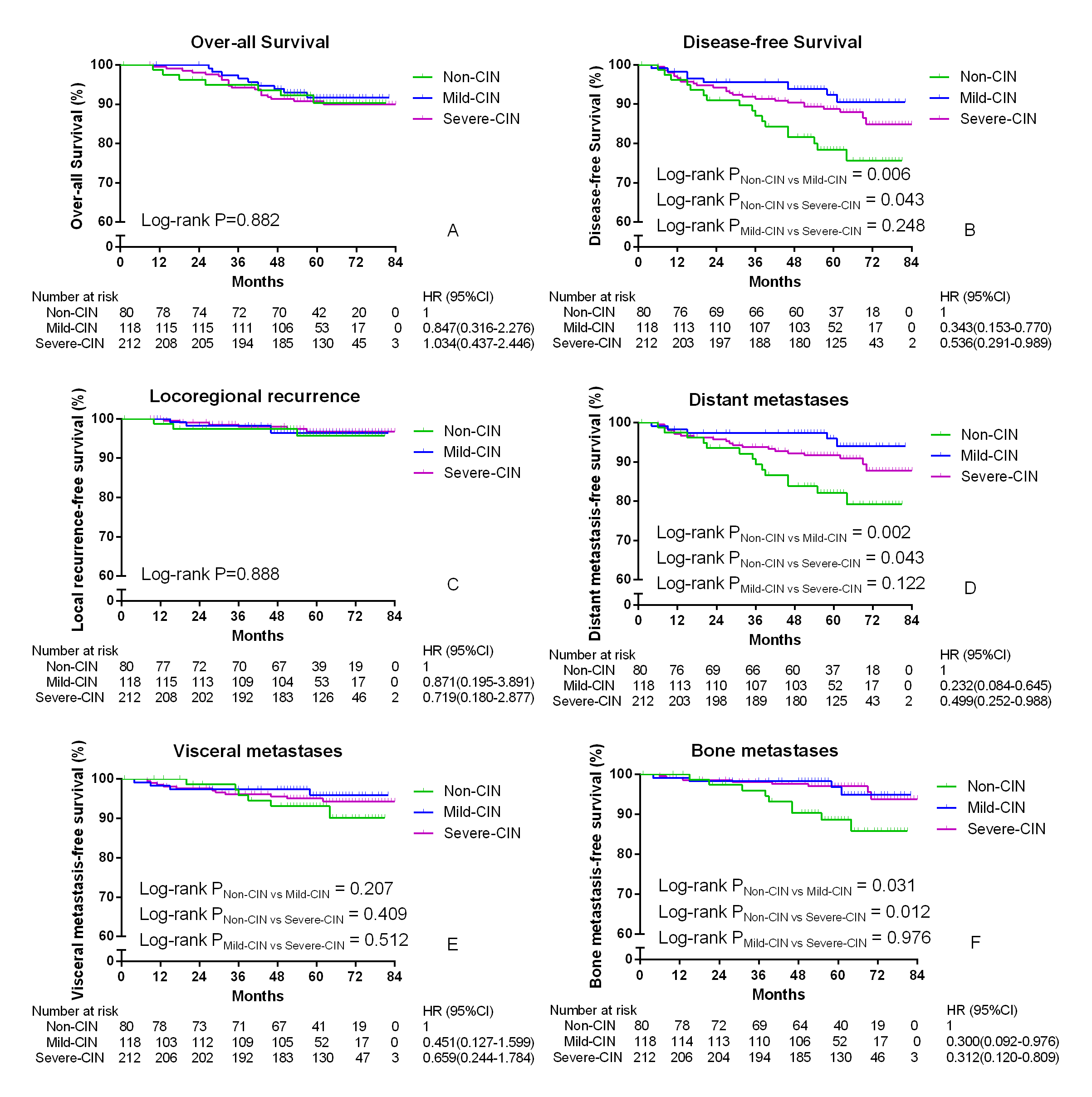


**Supplementary Table.1** Clinicopathologic characteristics of patients with different degrees of CIN

| Characteristic | Non-CIN  (n=80) | CIN (n=330) | | P value |
| --- | --- | --- | --- | --- |
|  |  | Mild (n=118) | Severe (n=212) |  |
| Age (year), median (range) | 50.5(33-74) | 51(28-82) | 51(25-76) | 0.734 |
| Menopausal status |  |  |  | 0.800 |
| Post-menopausal | 38(47.4%) | 60(50.8%) | 111(51.9%) |  |
| Pre-menopausal | 42(52.6%) | 58(49.2%) | 102(48.1%) |  |
| *≤40* | *14(17.8%)* | *41(34.7%)* | *72(34.0%)* | *0.884** |
| *>40* | *28(35.8%)* | *17(14.5%)* | *30(14.1%)* |  |
| Leukocytes, mean±SD | 7.15±1.78 | 6.36±1.47 | 6.33±1.62 | <0.001§ |
| Neutrophils, mean±SD | 4.56±1.33 | 4.04±1.18 | 3.84±1.37 | <0.001§ |
| Lymphocytes, mean±SD | 1.98±0.67 | 1.80±0.51 | 1.94±0.54 | 0.060§ |
| Platelet, mean±SD | 230.45±59.14 | 232.50±64.22 | 219.07±53.80 | 0.088§ |
| Pathological T staging |  |  |  | 0.757ǂ |
| pT1 | 37(46.3%) | 52(44.1%) | 94(44.3%) |  |
| pT2 | 41(51.2%) | 58(49.2%) | 107(50.5%) |  |
| pT3/4 | 2(2.5%) | 8(6.8%) | 11(5.2%) |  |
| Pathological N staging |  |  |  | 0.017ǂ |
| 0 | 52(65.0%) | 74(62.7%) | 115(54.2%) |  |
| 1-3 | 17(21.3%) | 30(25.4%) | 47(22.2%) |  |
| 4-9 | 7(8.8%) | 10(8.5%) | 29(13.7%) |  |
| ≥10 | 4(5.0%) | 4(3.4%) | 21(9.9%) |  |
| TNM staging |  |  |  | 0.005ǂ |
| Stage I | 28(35%) | 36(30.5%) | 50(23.6%) |  |
| Stage II | 40(50%) | 66(55.9%) | 109(51.4%) |  |
| Stage III | 12(15%) | 16(13.6%) | 53(25.0%) |  |
| Peritumoral vascular invasion |  |  |  | 0.105 |
| Absent | 76(95.0%) | 116(98.3%) | 197(92.9%) |  |
| Present | 4(5.0%) | 2(1.7%) | 15(7.1%) |  |
| ER status |  |  |  | 0.825 |
| Positive | 48(60.0%) | 70(59.3%) | 120(56.6%) |  |
| Negative | 32(40.0%) | 48(40.7%) | 92(43.4%) |  |
| PR status |  |  |  | 0.312 |
| Positive | 26(32.5%) | 49(41.5%) | 89(42.0%) |  |
| Negative | 54(67.5%) | 69(58.5%) | 123(58.0%) |  |
| HER-2 status |  |  |  | 0.117 |
| Positive | 24(30.0%) | 36(36.7%) | 85(36.7%) |  |
| Negative | 56(70.0%) | 82(63.3%) | 127(63.3%) |  |
| Type of surgery |  |  |  | 0.949 |
| Radical | 72(90.0%) | 105(89.0%) | 188(88.7%) |  |
| Conservative | 8(10.0%) | 13(11.0%) | 24(11.3%) |  |
| Chemotherapy regimens |  |  |  | <0.001 |
| FEC | 33(41.2%) | 32(27.1%) | 30(14.2%) |  |
| TC | 13(16.3%) | 8(6.8%) | 6(2.8%) |  |
| TEC | 34(42.5%) | 78(66.1%) | 176(83.0%) |  |
| * The P value was analyzed for the pre-menopausal women according to ages under 40 or elder.  § These P values were estimated by one-way ANOVA.  ǂ These P values were estimated by Spearman correlation analyses.  The rest P values were estimated by Wilcoxon rank-sum tests.  CIN = chemotherapy-induced neutropenia; SD = Standard Deviation; ER = Estrogen receptor; PR = Progesterone receptor; HER-2 = Human epidermal growth factor receptor-2; FEC = 5-fluorouracil/epirubicin/cyclophosphamide; TEC = docetaxel/epirubicin/cyclophosphamide; TC = docetaxel/cyclophosphamide. | | | | |
